# Associations between objective sleep measures and proteomic aging: The ARIC Study

**DOI:** 10.1093/geroni/igaf122.4073

**Published:** 2025-12-31

**Authors:** Chunyu Liu, Sanaz Sedaghat, Anna Prizment, Shuo Wang, Keenan Walker, Pamela Lutsey, Brion Maher, Adam Spira

**Affiliations:** Johns Hopkins Bloomberg School of Public Health, Baltimore, Maryland, United States; University of Minnesota, Minneapolis, Minnesota, United States; University of Minnesota, Minneapolis, Minnesota, United States; University of Minnesota, Minneapolis, Minnesota, United States; National Institute on Aging, Baltimore, Maryland, United States; University of Minnesota, Minneapolis, Minnesota, United States; Johns Hopkins Bloomberg School of Public Health, Baltimore, Maryland, United States; Johns Hopkins Bloomberg School of Public Health, Baltimore, Maryland, United States

## Abstract

Increasing evidence links poor sleep to accelerated biological aging; however, associations between sleep and proteomic age acceleration (PAC) remain understudied. We examined these associations among 1,554 participants (mean age 60.5±5.5 years, 99.0% White) in the Atherosclerosis Risk in Communities study (ARIC; waves 3 [1993-1995], 5 [2011-2013]) and the Sleep Heart Health Study (SHHS; waves 1 [1996]) who had polysomnography and proteomic data. Predictors were sleep-disordered breathing (SDB) and hypoxia, and sleep architecture, fragmentation, and duration (SHHS w1). Outcomes were proteomic age, derived from plasma (ARIC w3/w5; SomaScan v.4) using four published clocks (Wang, Sathyan, Tanaka, Lehallier). Linear regression assessed cross-sectional associations between sleep and PAC (ARIC w3); linear mixed models assessed associations between sleep and PAC longitudinal changes over time (ARIC w3 and w5). All models adjusted for age, race, education, study site, smoking, alcohol use, BMI, and sleep medication. Cross-sectionally, SDB (Wang: B = 0.59 [0.04, 1.14]; Sathyan: 0.91 [0.06, 1.76]), less REM sleep (Tanaka: -0.04 [-0.07, -0.01]; Lehallier: -0.03 [-0.06, -0.00] ), and lower sleep efficiency (Tanaka: -0.02 [-0.05, -0.00]) were associated with higher PAC. Longitudinally, lower REM oxyhemoglobin saturation (Sathyan: −0.27 [−0.45, −0.08]; Tanaka: −0.17 [−0.31, −0.03]), less slow-wave sleep (Tanaka: −0.03 [−0.05, −0.01]), higher arousal index (Tanaka: 0.03 [0.00, 0.05]), lower sleep efficiency (Wang: −0.05 [−0.09, −0.00]), and sleep > 8 h (Wang: 0.83 [0.05, 1.60]; Sathyan: 1.33 [0.35, 2.31]) were associated with increasing PAC (all p < 0.05). Findings suggest poor sleep accelerates proteomic aging, emphasizing sleep health as a target for aging-related disease prevention.

